# Influence of mentors’ innovation support on postgraduates’ proactive innovation behavior: the chain-mediating role of innovation efficacy and creative process engagement

**DOI:** 10.3389/fpsyg.2026.1737206

**Published:** 2026-04-29

**Authors:** Jinyang Wei, Haoping Peng, Yanfeng He, Chengyao Shuai, Anyang Wang

**Affiliations:** 1 School of Marxism, China University of Mining and Technology, Xuzhou, China; 2 School of Petroleum and Natural Gas Engineering, Changzhou, China

**Keywords:** chain-mediating role, creative process engagement, innovation efficacy, mentors’ innovation support, postgraduates’ proactive innovation behavior

## Abstract

**Objective:**

Postgraduates are the reserve force of national innovation-driven development, and enhancing postgraduates’ proactive innovation behavior has become the focus of higher education reform. This study focuses on the influence of mentors’ innovation support on postgraduates’ proactive innovation behavior and analyzes the internal conduction mechanism of innovation efficacy and creative process engagement.

**Methods:**

A questionnaire survey was conducted on 1200 postgraduates by a random sampling method, and data were collected using the mentors’ innovation support scale, proactive innovation behavior scale, innovation efficacy scale, and creative process engagement scale. Amos 24.0 and SPSS 26.0 statistical software were used to carry out correlation analysis and the chained mediation effect tests sequentially.

**Results:**

There is a significant correlation between mentors’ innovation support, innovation efficacy, creative process engagement, and postgraduates’ proactive innovation behavior. There are significant mediation effects and chain mediation effects between mentors’ innovation support and postgraduates’ proactive innovation behavior, in which the mediation effect of innovation efficacy is stronger than the mediation effect of creative process engagement and the chain mediation effect between the two.

**Conclusion:**

There is a relationship between mentors’ innovation support and postgraduates’ proactive innovation behavior, and this relationship is partially realized through the mediating effect of innovation efficacy and creative process engagement.

## Introduction

Postgraduate education serves as a crucial foundation for the country’s innovation-driven development and plays a vital role in cultivating high-level innovative talent and generating groundbreaking achievements (Zhang and Chen, 2023). As the scale of postgraduate education in China continues to expand, achieving a qualitative leap alongside quantitative growth has emerged as a central challenge in higher education reform (Gu et al., 2025). The rapid technological advancements and the reconfiguration of the global innovation landscape in the digital intelligence era necessitate that postgraduate students respond swiftly to environmental changes (Liu et al., 2023). Specifically, these students must proactively update research methodologies, optimize research processes, and innovate mechanisms and systems in alignment with the dynamic shifts in their environment and the evolving demands of scientific research problems (Fan et al., 2022). This proactive engagement is referred to as postgraduates’ proactive innovation behavior. Such behavior transcends the traditional passive response model of scientific research and serves as a key driving force behind the innovative vitality of research teams and university organizations (Fan et al., 2022; Chen et al., 2025).

Existing research has conducted multi-dimensional explorations around the influencing factors of postgraduates’ proactive innovation, forming two major research contexts: individual characteristics and external environment (Lee, 2025; Yu et al., 2025; Zhu et al., 2025). At the individual characteristic level, scholars have confirmed that sociodemographic variables, proactive personality, and innovative efficacy have significant predictive effects on innovative behavior (Cai, 2024). However, the occurrence of proactive innovative behavior is not an isolated individual act, but an interactive process embedded in a specific scientific research context, and its formation relies on external resource support and environmental empowerment (Zhou, 2024). At the external environment level, existing research mostly focuses on macro factors such as the guidance style of mentors, the organizational support atmosphere, and the supply of scientific research resources, but the specific mechanism of mentor support remains weak (Fang et al., 2024; Zhang and Zhao, 2025). In postgraduate education in China, the supervisor responsibility system is an important training model. As the guide of postgraduate students’ scientific research paths and an important provider of scientific research resources, the mentors’ innovation support has become a key variable influencing postgraduate students’ scientific research and innovation activities (Fan et al., 2022; Boonyarit, 2023). Organizational support theory posits that support from an organization can enhance an individual’s commitment and innovative behavior by addressing their emotional and resource needs (Kurtessis et al., 2017; Eisenberger et al., 2020). Mentors’ innovation support, as an informal type of organizational support, places greater emphasis on the emotional and psychological requirements of postgraduate students (Baranik et al., 2010; Israel et al., 2014). By fostering the expression of ideas, offering positive feedback, and creating platforms for innovation, this support combines both instrumental and emotional assistance, thereby igniting the intrinsic motivation for innovation among postgraduate students (McKinney et al., 2019; Subramanian et al., 2022). When these students perceive innovative support from their mentors, they are likely to develop a reciprocal intention to contribute, guided by the principle of mutual benefit in social exchange. Following the completion of fundamental research tasks, they are more inclined to exhibit proactive innovative behavior (Fan et al., 2019; Otor et al., 2026).

Although previous studies have briefly addressed the relationship between mentor support and innovative behavior, significant research gaps remain. First, most existing studies conceptualize mentor support as a unidimensional construct, failing to thoroughly analyze the functional pathway of “innovation support” and lacking an exploration of the internal logical chain connecting the two (Fan et al., 2022; Liu et al., 2024). Second, the investigation of intermediary mechanisms is insufficient, as the role of individual psychological cognitive factors in mediating the relationship between mentors’ innovation support and proactive innovation behavior has yet to be elucidated. Prior research has established that innovation efficacy, a central construct in social cognitive theory, reflects an individual’s subjective belief in their capacity to achieve innovation goals and can significantly enhance the likelihood of engaging in proactive innovation behaviors (Li K. J. et al., 2025; Ma and Zhou, 2025; Sun et al., 2025). Furthermore, creative process engagement, characterized as a focused, continuous, and positive psychological state in scientific research, acts as a crucial link between individual cognition and innovative behavior (Begum et al., 2022). This investment is positively influenced by self-efficacy and subsequently affects the performance of innovative behavior (Khan and Abbas, 2022). Therefore, innovation efficacy and creative process engagement may create a mediating chain linking mentors’ innovation support to postgraduates’ proactive innovation behavior. This mechanism warrants urgent empirical investigation (Chou et al., 2024).

In view of this, innovation efficacy and creative process engagement are introduced to clarify the role of mentors’ innovation support on postgraduates’ proactive innovation behavior, especially to verify the chain mediating role of innovation efficacy and creative process engagement. The research will use a mature scale to construct a questionnaire, take postgraduate students as the survey objects, and use SPSS 26.0 and AMOS 24.0 software for data analysis to empirically test the research model and help understand the influencing factors of postgraduates’ proactive innovative behavior, with a view to providing enlightenment and suggestions for high-quality training of postgraduates in colleges and universities.

## Theory and hypotheses

### Mentors’ innovation support and postgraduates’ proactive innovation behavior

Existing research mostly defines the connotation of individual innovative behavior from a process perspective. The structure of innovative behavior has been divided into various models, such as three-stage, five-stage, and two-stage frameworks. For example, Scott and Bruce (1994) believe that innovative behavior includes three stages: innovative conception, seeking support for the idea, and “productization” of innovative ideas. Nevertheless, their empirical research indicates the presence of only a single dimension. Kleysen and Street (2001) summarized innovative behavior into five stages: finding opportunities, generating ideas, forming investigations, supporting, and applying. However, the results of their empirical validity tests are not satisfactory. Empirical testing of the five-stage perspective on innovative behavior reveals that an individual’s innovative behavior can be categorized into two distinct stages: the generation of innovative ideas and the implementation of creative concepts (Krause, 2004; Yuan and Woodman, 2010). Analysis of the empirical test results conducted by Chinese researchers indicates that this two-stage framework for understanding innovative behavior possesses significant practical applicability (Zhao et al., 2014).

Innovative behavior can be categorized into active and passive forms based on differing behavioral motivations (Zhou et al., 2024). Passive innovative behavior is characterized as involuntary and misaligned with an individual’s cognition, often compelled by pressures from the organizational environment (Heidenreich and Kraemer, 2015). Based on the varying self-construction states of scientific researchers, passive innovation behaviors are categorized into three types: perfunctory innovation, expedient innovation, and compliant innovation (Zhao et al., 2015). First, perfunctory innovation describes a scenario in which an individual is hesitant to engage in the innovations promoted by organizational norms but feels compelled to adhere to these constraints. Consequently, the individual adopts superficial measures to meet the innovation requirements set forth by the organization (Zou et al., 2025). Second, expedient innovation occurs when individuals consider both their personal understanding and the demands of organizational norms, allowing them to pursue innovations that align with these standards (Cheng et al., 2025). Finally, compliant innovation involves an individual’s complete dedication to the innovation activities endorsed by the organization, resulting in a mechanical execution of the prescribed innovation directives (van den Broek and van Veenstra, 2018; Doyle et al., 2019). In contrast, proactive innovative behavior denotes voluntary actions driven by intrinsic motivation, wherein individuals actively prepare for future innovations and address challenges encountered in the innovation process. In the context of postgraduate studies, active innovative behavior among postgraduates can be defined as the process in which they actively seek original ideas and novel methods during academic research activities, demonstrating a willingness to assume risks in pursuit of beneficial innovation outcomes. This behavior encompasses three dimensions: spontaneity, preliminary preparation, and the ability to overcome obstacles (Zhao et al., 2014).

Spontaneity denotes the psychological inclination of individuals to engage voluntarily and actively in innovative activities without external compulsion. It arises, in part, from postgraduates’ intrinsic interest and curiosity in scientific research, which is evident in their ability to identify research problems and their desire to address these issues through novel approaches (Deci and Ryan, 2008). Additionally, external incentives, team dynamics, social recognition, and other factors can transform attitudes toward innovation from passive to active, facilitating the internalization of external motivation (Huynh et al., 2019; Lee et al., 2022; Norena-Chavez and Sosa Varela, 2025). This spontaneity serves as the psychological foundation for postgraduates’ proactive innovative behavior. Preliminary preparation refers to the planning and resource preparation made by individuals in order to effectively propose and implement innovative ideas. In view of the inherent ambiguity and uncertainty of innovative activities, postgraduates need to have a certain degree of forward-looking awareness, clarify goals, anticipate difficulties, and make corresponding arrangements before taking action. Specifically, it includes two aspects: first, mental preparation, which refers to grasping research needs and forming novel and feasible research ideas by consulting experts, peers, and mentors; second, resource preparation, which refers to systematically supplementing knowledge, collecting literature, preparing equipment and funds, and providing necessary support for the innovation process (Li and Wu, 2011; Odoardi, 2015).

Overcoming obstacles pertains to the actions of postgraduates who are resolute in addressing challenges that emerge during the innovation process while maintaining their commitment to achieving success. Innovation frequently entails intricate challenges and the potential for failure. Consequently, postgraduates must possess considerable mental resilience and perseverance to effectively tackle problems. They must also demonstrate tenacity in the face of setbacks, thereby facilitating progress in innovation through persistent efforts (Fan et al., 2022; Fang et al., 2024). The leader’s personality characteristics and leadership style will affect individual innovative behavior (Lan et al., 2021; Liu et al., 2026). In terms of the impact of leaders on subordinates’ responsibilities, some scholars have discussed the impact of leadership style on innovative behavior. Among them, the inclusive leadership style lies in its “inclusiveness.” Inclusive leadership refers to a leadership that pays attention to the individual differences of postgraduates, encourages postgraduates to express different opinions, is good at listening to postgraduates’ opinions, and provides support and resources to postgraduates (Huang et al., 2025; Katsaros, 2025). Transformational leadership style emphasizes “reformation.” Transformational leadership is defined as making postgraduates aware of the importance of the tasks they undertake, stimulating the high-level needs of subordinates, establishing an atmosphere of mutual trust, and prompting subordinates to sacrifice their own interests for the benefit of the organization and achieve results that exceed original expectations (Xin et al., 2024; Li X. Y. et al., 2025). Different from other leadership styles, leadership innovation support means that leaders give full recognition and support to subordinates’ innovative behaviors and activities, and provide necessary resources.

In the academic careers of postgraduates, mentors maintain the closest contact with them (Stravakou and Lozgka, 2022; King, 2024). Mentors’ innovation support serves as a crucial situational factor influencing postgraduates’ scientific research and innovation activities, significantly affecting their innovative behavior. This support emphasizes the emotional needs of postgraduates, encourages them to articulate their ideas, provides constructive feedback, and fosters innovative thinking (Li Q. et al., 2025). Organizational support theory posits that an organization’s attention to and support for its members are critical factors that encourage individuals to remain with the organization and actively contribute to its success (Yu and Frenkel, 2013; Wang, 2024). Firstly, mentors convey trust and recognition through emotional support, affirming postgraduates’ innovative capabilities in a timely manner. This affirmation enhances their self-confidence and sense of security, motivating them to concentrate more on scientific research practices and facilitating the generation of innovative outcomes (Hu, 2017; Li Q. et al., 2025). Secondly, mentors establish a material foundation for innovative activities by supplying venues, equipment, and other instrumental resources. They assist postgraduates in clarifying their research directions and guide them in proactively seeking and integrating innovative resources (Xiang et al., 2023). Finally, through positive feedback and guidance, mentors aid postgraduates in identifying problems, adjusting their research directions, and gradually improving their autonomy in scientific inquiry as well as their ability to overcome obstacles to innovation (Cutter et al., 2025; Nwalieji et al., 2025). Based on this analysis, this study proposes the following hypotheses:

*Hypothesis 1:* Mentors’ innovation support positively affects postgraduates’ proactive innovation behavior.

### The mediating role of innovation efficacy

According to social cognitive theory, an individual’s cognitive factors significantly influence their engagement with the social environment (Bandura, 1989). In the realm of higher education, mentor innovation support denotes postgraduates’ perception of the emotional care and support offered by their mentors in innovation-related endeavors (Li Q. et al., 2025). Drawing from creativity theory, Tierney and Farmer (2002) integrated self-efficacy into innovation research, defining innovation efficacy as an individual’s belief in their capacity to generate and actualize innovative ideas successfully. Innovation efficacy is shaped not only by personal skills and experience but also by the external environment’s support and ambiance. In academic research, the relationship between mentors and postgraduates is typically intimate. Providing timely emotional and psychological support can assist postgraduates in cultivating a positive psychological outlook, diminishing self-blame and frustration following setbacks in scientific research and innovation (Zou et al., 2023; Shan and Rasool, 2025). The accrual of such positive psychological resources can enhance postgraduates’ innovative efficacy. Simultaneously, mentors establish a robust groundwork for postgraduate students’ innovative endeavors by furnishing cutting-edge experimental facilities, specialized academic instruction, project resources, and other tangible support (Li K. J. et al., 2025). These actions bolster students’ confidence in accomplishing innovative tasks, ultimately elevating their innovation efficacy.

Social cognitive theory posits that beliefs significantly influence an individual’s cognition, attitudes, and behaviors (Bandura, 1991). Innovative efficacy offers essential psychological support to postgraduate students facing setbacks and challenges, motivating them to respond with positivity and optimism. When postgraduate students hold positive beliefs, they are more likely to proactively engage in sustained practice, creatively address technical difficulties, and unlock their innovative potential, thereby fostering continuous breakthroughs in their innovation activities until they achieve success (Zhang et al., 2024). Research indicates that the perception of innovation efficacy acts as both an incentive and a support mechanism in the realms of scientific research innovation and the development of creativity, thereby holding significant value for decision-making processes (Takeed et al., 2025). Whether this sense of efficacy is derived from actual innovation outcomes or bolstered by mentor support, it aids postgraduates in developing a positive self-perception. This, in turn, cultivates greater frustration resistance and psychological resilience, thereby improving the quality of innovative output and ultimately establishing a virtuous cycle of “results output - efficacy feedback - results output”. Based on this analysis, this study proposes the following hypotheses:

*Hypothesis 2:* Innovation efficacy plays a mediating role between mentors’ innovation support and postgraduates’ proactive innovation behavior.

### The mediating role of creative process engagement

Creative process engagement refers to the degree of individual investment in the innovation process, including cognitive processes such as problem identification, information search, and idea generation. As explained by Reiter-Palmon and Illies (2004), creative problem-solving requires extensive and in-depth cognitive processing. Social exchange theory (Thibault, 1959) posits that resources are objects to be exchanged, offering a theoretical foundation for understanding interactive and cooperative relationships within educational systems. Individuals and groups establish and maintain stable social connections by exchanging resources, emotions, and identities, thereby maximizing their own interests (Cropanzano and Mitchell, 2005). According to the social exchange theory, the teacher-student relationship represents a distinctive exchange dynamic (Ibidunni et al., 2021). When mentors offer emotional nurturing and practical assistance to postgraduates, the students develop a heightened feeling of inclusion and backing, leading them to reciprocate by demonstrating positive academic dedication and task engagement. Favorable teacher-student interactions foster a constructive innovative environment, thereby motivating postgraduates to actively participate in the innovation process (Jiang et al., 2025). First, the mentor stimulates the intrinsic motivation of postgraduates by providing positive emotional resources, prompts them to take the initiative to work, comprehensively connect with information resources, contribute innovative ideas, and give back to the guidance with excellent results. Secondly, the care and support of the mentor for postgraduates, as well as the tolerance of innovation failure, help alleviate work burnout, enable postgraduates to focus on their work, and ultimately contribute to the achievement of goals. Finally, the instrumental support provided by the mentors helps postgraduates obtain more resources, proactively meet challenges, comprehensively identify problems, formulate practical plans, and complete research tasks.

Empirical research showed that creative process engagement has a significant promotion effect on proactive innovation behavior (Chen et al., 2022). Creative process engagement reflects the flexibility of postgraduates’ cognitive paths, manifested in the allocation of attention to a certain aspect of the task and the emphasis they place on solution exploration, which helps to promote proactive innovative behavior of postgraduates (Rubenstein et al., 2018; Karwowski and Beghetto, 2019). Zhang and Bartol (2010) found that when individuals focus on problem identification, extensively search for information resources, and actively seek solutions, novel solution ideas and solutions often emerge. When mentors provide instrumental and emotional support to postgraduates, an exchange relationship is formed between mentors and postgraduates, creative process engagement is significantly enhanced, and the intrinsic motivation is effectively stimulated, thus continuously promoting the generation of their proactive innovative behaviors. Based on this analysis, this study proposes the following hypotheses:

*Hypothesis 3:* The creative process engagement plays a mediating role between mentors’ innovation support and postgraduates’ proactive innovation behavior.

### The chain mediating effect of innovation efficacy and creative process engagement

Self-efficacy can effectively promote individuals to actively participate in the innovation process, thereby stimulating the emergence of innovative behaviors (Mahmood et al., 2019; Mirza et al., 2024). From the perspective of the mechanism of action, innovation efficacy, as a kind of positive psychological capital, can guide postgraduates to fully explore their potential and integrate external resources according to specific task requirements, actively explore ways to solve problems, and devote themselves wholeheartedly to innovative activities (Puente-Díaz, 2016). Efficacy beliefs indirectly affect activity participation by affecting positive factors (Rubenstein et al., 2018). Individuals with higher levels of self-efficacy are confident, have the courage to face challenges, and are willing to invest more time and energy in the innovation process. On the contrary, individuals with a low level of self-efficacy lack confidence in their own innovative abilities, hold a negative mentality, and are less likely to participate in creative processes or related activities, and are more likely to choose negative behavioral reactions such as avoidance (Yuan et al., 2023; Xiang et al., 2024). Tierney and Farmer (2002) proposed that innovation efficacy pertains to an individual’s capacity and assurance in producing innovative behaviors within organizational contexts, representing a specialized expansion and utilization of self-efficacy within the realm of innovation. Research shows that the higher the level of innovation efficacy, the more significant the positive promotion effect on creative process engagement (Li et al., 2021; Tantawy et al., 2021). Based on this, this study proposes the following hypotheses:

*Hypothesis 4:* Innovation efficacy and creative process engagement play a chain mediating role between mentors’ innovation support and postgraduates’ proactive innovation behavior.

Based on the previous analysis, the mentors’ innovation support first improves the innovation efficacy, and then promotes the formation of postgraduates’ proactive innovation behavior by influencing the creative process engagement. Thus, we predict that (1) the mentors’ innovation support can have a positive impact on postgraduates’ proactive innovative behaviors. (2) Innovation efficacy will mediate the relationship between mentors’ innovation support and postgraduates’ proactive innovation behaviors. (3) Creative process engagement will mediate the relationship between mentors’ innovation support and postgraduates’ proactive innovation behaviors. (4) Mentors’ innovation support influences postgraduates’ proactive innovative behavior through the chain mediation of innovation efficacy and creative process engagement.

## Materials and methods

### Research subjects and data collection

From June to July 2025, data collection was conducted via an online questionnaire using the Questionnaire Star platform. This research employed a random sampling method, selecting postgraduates from six universities located in Changzhou, Xuzhou, Zhenjiang, and Nanjing. A total of 100 students from each university were invited to complete the questionnaire to enhance the accuracy and generalizability of the findings. The selected institutions comprised “double-first-class” universities and other ordinary universities. Among them, double-first-class universities include first-class university construction plan grouped into Category A or B, and first-class discipline construction universities. Each questionnaire was accompanied by a comprehensive informed consent agreement that clearly outlined the study’s purpose, emphasized the voluntary nature of participation, and assured the anonymity and confidentiality of the data collected.

A total of 1,200 questionnaires were distributed and collected, yielding 1,106 responses. After manual screening, 987 valid questionnaires were retained, resulting in an effective response rate of 82.25%. The demographic characteristics of the sample are as follows: females represent 42.86%, and males represent 57.14% of the gender distribution. In terms of grade distribution, first-year master’s students comprise 30.19%, second-year master’s students also account for 32.62%, while third-year master’s students and doctoral students together constitute 37.18%. Regarding the school category, double-first-class universities make up 50.46%, while non-double-first-class universities account for 49.54%. In terms of subject distribution, science and engineering represent 35.66%, management comprises 29.28%, and literature, history, and philosophy account for 35.06%.

### Variables

To improve the reliability and validity of the survey questionnaire, this research used a validated maturity scale. The items of the questionnaire were designed in accordance with Likert’s 5-point scale, assigning 1–5 points, respectively, from the degree of “completely inconsistent” to that of “completely consistent.” The Appendix provides the specific item texts of the four primary measurement tools.

### Mentors’ innovation support

Mentors’ innovation support was assessed using 5 items closely related to innovation activities. These items were selected from the supportive leadership questionnaire developed by Tierney and Farmer (2004) and were adapted to align with the graduate research context. For instance, statements included, “To foster my creative endeavors, my mentor diligently provides the necessary resources” and “My mentor has publicly acknowledged my innovative efforts.” In this study, the Cronbach’s *α* for the scale was 0.902.

### Innovation efficacy

This variable was measured by the scale developed by Carmeli and Schaubroeck (2007). We have rephrased the original entries to align with the research context concerning the innovative abilities of postgraduate students. There were altogether 8 items, including the items “I can be creative when I finish my set goals” and “I am confident in my creativity across many different tasks.” In this study, the Cronbach’s *α* for the scale was 0.935.

### Creative process engagement

This variable was measured by the scale developed by Zhang and Bartol (2010) with a total of 11 items. The scale includes three dimensions: problem identification, information search and coding, and idea generation, with 3, 3, and 5 items, respectively. The higher the value, the more postgraduates invest in the innovation process. For example, it contained the question item “I spend considerable time trying to understand the nature of the problem.” In this study, the Cronbach’s *α* for the scale was 0.918.

### Postgraduates’ proactive innovation behavior

This variable was assessed with a 16-item scale developed by Zhao et al. (2014). The items were adapted to reflect proactive innovation in academic research rather than organizational settings. For instance, it contained the question items “To solve the problem, I took the initiative to put forward suggestions” and “I proactively seek out new ideas to improve work processes or products.” In this study, the Cronbach’s α for the scale was 0.934.

### Control variables

To control for potential confounding effects, this study included gender, disciplinary field, and education level as control variables in the analysis (George and Zhou, 2007). Gender disparities can impact students’ self-efficacy, subsequently affecting their academic performance and executive function (Wang and Yu, 2023). Studies indicate that females tend to demonstrate higher self-efficacy levels in language-related domains like literature, whereas males tend to excel in technology and science (Cui et al., 2024; Moraga-Pumarino et al., 2025). Educational attainment can significantly influence an individual’s attitude toward sustainable innovation (Loučanová et al., 2023). The educational attainment of respondents reflects their knowledge base, innovative awareness, cognitive abilities, and the social networks and capital that shape their innovative behavior (Quazi and Talukder, 2011). Research indicates that individuals with higher educational levels exhibit greater sensitivity to the issues surrounding sustainable innovation (Zhang et al., 2022). By controlling these variables, the study aims to provide a more precise estimation of the influence of independent variables on dependent variables, thereby enhancing the internal validity and theoretical explanatory power regarding postgraduate students.

## Results

### Reliability analysis

The Cronbach’s α coefficient and composite reliability were calculated using SPSS26.0 to test the reliability of the questionnaire. The results are shown in Table 1. When the Cronbach’s α coefficient approaches or exceeds 0.9, it can be considered that the reliability of the measuring tool is very high. The Cronbach’s α values of the variable scales such as mentors’ innovation support, innovation efficacy, creative process engagement, and postgraduates’ proactive innovation behavior were all above 0.9. Meanwhile, the composite reliability (CR) values were 0.907, 0.939, 0.954, and 0.957 respectively, all meeting the reliability standards required by statistics, indicating that the four main variable scales in this questionnaire all have a relatively high reliability level.

**Table 1:** Reliability and validity analysis of variables.

Variables	Factor load	Cronbach’s *α*	CR	AVE
Mentors’ innovation support	0.612–0.924	0.902	0.907	0.664
Innovation efficacy	0.723–0.922	0.935	0.939	0.658
Creative process engagement	0.692–0.943	0.918	0.954	0.658
Postgraduates’ proactive innovation behavior	0.644–0.893	0.934	0.957	0.614

### Validity analysis

Validity includes content validity and structural validity. All the options in this questionnaire are derived from mature scales in the literature and have been modified with reference to classic literature of research value. Therefore, it performs exceptionally well in terms of content validity. Structural validity is divided into convergent validity and discriminative validity. Convergent validity mainly focuses on the correlation among different measurement methods. AVE is the mean variance extract, which represents the proportion of the total variation of the variable explained by each factor. The larger the AVE value, the greater the proportion of variance extracted by this factor and the higher the validity. CR stands for composite reliability, which is an important indicator for measuring the internal consistency of a factor. As shown in Table 1, AVE is all greater than 0.6, and CR is all greater than 0.9, indicating that the questionnaire possesses good convergent validity.

The discriminant validity test was conducted on four variables, namely mentors’ innovation support, innovation efficacy, creative process engagement, and postgraduates’ proactive innovation behavior, with the help of the software Amos 24.0 and the confirmatory factor analysis method. The results were shown in Table 2. The x^2^/df of the four-factor model was 2.654, lower than the limited numerical value 3. Its RMSEA was 0.064, below the numerical value 0.08. Its CFI was 0.977, TLI was 0.969, and NFI was 0.966, all of which exceeded the threshold of 0.9. From the above data, the fitting indicators of the four-factor model were better than the evaluation criteria of the fitting indicators. In view of this conclusion, it can be said that the four-factor model of mentors’ innovation support, creative process engagement, innovation efficacy, and postgraduates’ proactive innovation behavior had a good fitting effect. The four variables with good discriminant validity were independent of each other, they can be utilized for subsequent research and analysis.

**Table 2:** The results of the competitive model’s validation factor analysis.

Variables	x^2^/df	RMSEA	CFI	TLI	NFI
Evaluation criteria of the indicator	<3	<0.08	>0.9	>0.9	>0.9
One-factor model	34.535	0.237	0.776	0.702	0.772
Two-factor model	19.519	0.176	0.886	0.835	0.876
Three-factor model	12.221	0.137	0.933	0.900	0.928
Four-factor model	2.654	0.064	0.977	0.969	0.966

One-factor model: mentors’ innovation support + innovation efficacy + creative process engagement + postgraduates’ proactive innovation behavior. Two-factor model: mentors’ innovation support, innovation efficacy + creative process engagement + postgraduates’ proactive innovation behavior. Three-factor model: mentors’ innovation support, innovation efficacy, creative process engagement + postgraduates’ proactive innovation behavior. Four-factor model: mentors’ innovation support, innovation efficacy, creative process engagement, postgraduates’ proactive innovation behavior.

### Homologous variance test

To control the homologous variance, methods such as filling the questionnaire anonymously, keeping the items secret and setting inverse questions were adopted to improve the authenticity of the data. Harman’s single factor test was used to select variables with eigenvalues higher than 1, with the help of SPSS 26.0 for principal cause analysis. The results showed that a total of four factors with eigenvalues higher than 1 were found, and the explanatory variance of the first principal component accounted for 35.59% of the total variance, which was much lower than the recommended value of 50%. Therefore, it can be considered that the homogeneity variance of the scale data was not significant, and further research can be conducted.

### Descriptive analysis

The means, standard deviations, and Pearson correlation coefficients among the main variables and their respective dimensions are presented in Table 3. The correlation coefficients of the four variables in this study are less than 0.7, indicating that there is no serious multicollinearity problem among the major variables. Moreover, it can be concluded that there was a significant correlation between the four variables of mentors’ innovation support, innovation efficacy, creative process engagement, and postgraduates’ proactive innovation behavior. The result was consistent with the research hypothesis, providing a basis for subsequent research on the relationship between the four variables.

**Table 3:** Means, SDs and correlation coefficients of the major research variables.

Variables	M	SD	1	2	3	4
1. Mentors’ innovation support	4.21	0.756	1			
2. Innovation efficacy	3.88	0.715	0.274**	1		
3. Creative process engagement	3.72	0.610	0.269**	0.575**	1	
4. Postgraduates’ proactive innovation behavior	3.86	0.628	0.409**	0.651**	0.639**	1

** indicates a significant correlation at the 0.01 level (double tailed).

### Mediation analysis

1. Main effect test. Linear regression method was used to test the direct impact of mentors’ innovation support on postgraduates’ proactive innovation behavior. Model M1 was constructed to carry out regression analysis by incorporating control variables such as graduates’ gender, discipline category, and school category into the equation. The results indicated that mentors’ innovation support had a significant positive impact on postgraduates’ proactive innovation behavior (*β* = 0.425, *p* < 0.01), confirming Hypothesis 1.
2. Mediation effect test. Innovation efficacy and creative process engagement were added as independent mediating variables between mentors’ innovation support and postgraduates’ proactive innovation behavior. The Model M2 and M3 were constructed, using the Bootstrap method to analyze the independent mediating effects of the two variables by repeating 5,000 sampling tests. The results showed that in model M2, mentors’ innovation support had a significant positive effect on innovation efficacy (*β* = 0.32, *p* < 0.01), while innovation efficacy had a significant positive effect on postgraduates’ proactive innovation behavior (*β* = 0.74, *p* < 0.01). Moreover, innovation efficacy played a partial mediating role. Based on these results, it can be said that hypothesis 2 was proven. In Model M3, mentors’ innovation support had a significant positive effect on creative process engagement (*β* = 0.36, *p* < 0.01), while creative process engagement had a significant positive effect on postgraduates’ proactive innovation behavior (*β* = 0.71, *p* < 0.01). Additionally, creative process engagement played a partial mediating role. Hypothesis 3 was proven by these findings.

Next, the chain mediating effect of innovation efficacy and creative process engagement was examined. The Bootstrap method was adopted, repeating sampling 5,000 times. The results of the effect analysis are shown in Table 4.

**Table 4:** Pathway and effect analysis of the impact of mentors’ innovation support on postgraduates’ proactive innovation behavior.

Effect pathway	Normalized effect value	Effect size	95% confidence interval	
			Lower limit	Upper limit
Pathway 1: Mentors’ innovation support—innovation efficacy—postgraduates’ proactive innovation behavior	0.165	38.8%	0.107	0.233
Pathway 2: Mentors’ innovation support—creative process engagement—postgraduates’ proactive innovation behavior	0.047	11.1%	0.017	0.091
Pathway 3: Mentors’ innovation support—innovation efficacy—creative process engagement—postgraduates’ proactive innovation behavior	0.073	17.2%	0.038	0.123
Total mediation effect	0.285	67.1%	0.199	0.371
Direct effect	0.140	32.9%	0.059	0.229
Total effect	0.425	100%	0.313	0.513

Through model path testing analysis, it was found that the total mediating effect was the sum of the effects of the three mediating paths. The value of it was 0.285, and its 95% confidence interval was [0.199, 0.371], excluding 0, which proved that the effect was significant. The total effect value of mentors’ innovation support on postgraduates’ proactive innovation behavior was 0.425, and its 95% confidence interval was [0.313, 0.513], excluding 0. The effect was significant, further validating hypothesis 1. The mediating effect values of innovation efficacy and creative process engagement between mentors’ innovation support and postgraduates’ proactive innovation behavior were 0.165 and 0.047, respectively. The former had a 95% confidence interval of [0.107, 0.233], while the latter had a 95% confidence interval of [0.017, 0.091], both excluding 0. The effect was significant, further supporting hypothesis 2 and hypothesis 3. The chain mediating effect value of innovation efficacy and creative process engagement in mentors’ innovation support and postgraduates’ proactive innovation behavior was 0.073, with a 95% confidence interval of [0.038, 0.123], excluding 0. Such an effect was significant. It demonstrated that mentors’ innovation support could positively affect innovation efficacy. Moreover, through the positive enhancement of creative process engagement, postgraduates’ proactive innovation behavior could be promoted. Based on these findings, hypothesis 4 was proven. At the same time, this study drew a standardized path coefficient graph for the structural equation of the chain mediating model, which visually displayed the chain mediating effect of innovation efficacy and creative process engagement between mentors’ innovation support and postgraduates’ proactive innovation behavior.

## Discussion

This study develops a chain mediation model to examine the positive impact of mentors’ innovation support on postgraduates’ proactive innovation behavior. Furthermore, innovation efficacy and creative process engagement serve as chain mediators in this relationship. The specific conclusions are as follows:

First, the mentors’ innovation support can have a positive impact on postgraduates’ proactive innovative behaviors.

Mentors’ innovation support, as an informal support mechanism, integrates a composite resource supply model that merges emotional and instrumental support (Lan et al., 2021; Liu et al., 2026). Emotional support effectively mitigates research anxiety among postgraduate students and enhances their innovation efficacy by fostering an inclusive and open academic environment, promoting critical thinking, and offering constructive feedback (Hosseini et al., 2024). In contrast, instrumental support supplies essential material resources for postgraduate students’ innovative endeavors through avenues such as connecting them with scientific research resources, establishing interdisciplinary research platforms, and imparting innovative methodologies (Hur et al., 2024). This dual support mechanism serves as a crucial catalyst for postgraduate students’ proactive engagement in innovative behavior, enabling them to achieve higher levels of innovation and participate more actively in innovative activities. Sufficient instrumental support alleviates resource limitations during the innovation process, empowering postgraduate students to propose and execute innovative ideas. According to social exchange theory, mentors’ innovation support represents a form of resource input originating from the organizational level. When postgraduate students recognize the innovative support provided by their mentors, they are motivated to reciprocate, thereby transforming the mentors’ support into an intrinsic driving force for proactive innovation. Consequently, following the completion of fundamental scientific research tasks, they are inclined to engage in innovative behaviors.

Second, innovation efficacy plays a significant mediating role between mentors’ innovation support and postgraduates’ proactive innovation behaviors.

Innovation efficacy refers to an individual’s belief in their ability to generate novel and valuable ideas (Tierney and Farmer, 2002). Individuals with a higher sense of innovation efficacy generally exhibit greater creativity and are confident in their ability to generate new ideas and support those ideas. In postgraduate academic activities, innovative behavior is mainly reflected in the generation and submission of innovative ideas. Graduate students’ sense of innovation efficacy will motivate them to engage in creativity-related activities. Research shows that there is a significant positive relationship between creative efficacy and innovative behavior, a conclusion that has been empirically supported in multiple educational scenarios (Chung et al., 2022; Fu et al., 2025). During graduate training, mentors’ innovation support plays a crucial role as an organizational contextual factor. This support aids in boosting postgraduates’ self-confidence and improving their innovation efficacy through offering experiential feedback, presenting role models, and regulating their psychological well-being (Su et al., 2019). Those postgraduates with elevated levels of innovation efficacy demonstrate confidence in their innovative skills and exhibit resilience in confronting obstacles and difficulties. Consequently, they are more inclined to leverage available supportive resources and participate actively in innovative endeavors, thereby fostering a culture of proactive innovative behavior among postgraduates.

Thirdly, creative process engagement mediates the relationship between mentors’ innovation support and postgraduates’ proactive innovation behaviors.

Several creativity theorists emphasize that a comprehensive understanding of the mechanisms underlying innovative achievements requires allocating greater research attention to the creative process itself (Mirza et al., 2024; Yang et al., 2025). Gilson and Shalley (2004) focused on the stages of idea generation and the development of alternative solutions in the innovation process, initially exploring the potential factors influencing individual participation. This study provides a novel perspective for advancing creativity theory by conceptualizing the input of the innovation process as a more comprehensive concept. This broader framework not only includes the stage of idea generation but also integrates cognitive elements such as problem identification, information search and coding, and idea generation (Karwowski and Beghetto, 2019).

According to social exchange theory (Madison et al., 2025; Nutsugah et al., 2025), mentors’ innovation support can effectively strengthen the relationship between mentors and postgraduates, enhance postgraduates’ concentration and enthusiasm, and encourage them to engage more actively with the information provided by mentors. Ultimately, it facilitates the transformation of innovative achievements. Mentors assist postgraduates in systematically identifying research problems and developing feasible research plans by offering instrumental resources such as information and equipment. Additionally, the emotional support from mentors can stimulate postgraduates’ intrinsic motivation, prompting them to immerse themselves in scientific research. The creative process engagement is crucial to this dynamic. Through deep engagement in problem exploration, scheme construction, and implementation, postgraduates convert the resource support and emotional encouragement provided by mentors into sustained and intentional innovative behaviors. This process not only enhances the effectiveness of mentors’ innovation support but also significantly fosters the development of postgraduates’ proactive innovative behaviors.

Fourth, innovative efficacy and creative process engagement play a chained mediating role between mentors’ innovation support and postgraduates’ proactive innovative behaviors.

Mentors’ innovation fosters an enhancement in postgraduates’ innovation efficacy, which in turn positively influences creative process engagement. This relationship subsequently amplifies postgraduates’ proactive innovative behaviors. Innovation efficacy and creative process engagement serve as chain intermediary variables within the framework of mentors’ innovation support and its impact on postgraduates’ proactive innovative behaviors. This paper constructs a chain model encompassing mentors’ innovation support, innovation efficacy, creative process engagement, and postgraduates’ proactive innovative behaviors, thereby elucidating the multiple pathways through which mentors’ innovation support influences postgraduates’ proactive innovative behaviors. A more comprehensive analysis of the mechanisms underlying postgraduate students’ proactive innovative behaviors reveals that those with high innovation efficacy are more inclined to tackle challenging research tasks, pursue innovative solutions, and independently advance the research process.

## Conclusion

This study investigates the relationship between mentors’ innovation support and the active innovation behavior of postgraduate students. Mentors’ innovation support, as an informal form of organizational assistance, significantly improves the innovative efficacy of postgraduate students through a model that integrates emotional and instrumental support. When postgraduate students recognize the innovative support provided by their mentors, they are motivated to reciprocate, in accordance with the principle of social exchange. This reciprocation leads them to internalize the mentors’ innovation support as an intrinsic motivator for active innovation, thereby increasing their commitment to the innovation process and fostering engagement in innovative exploration behaviors. First, mentors’ innovation support affects active innovation behavior through both innovation efficacy and creative process engagement. Among these two sequential mediating roles, the influence of innovation efficacy on proactive innovation behavior is significantly more pronounced than that of creative process engagement. Second, innovation efficacy and creative process engagement exhibit a chain mediating effect in the relationship between mentors’ innovation support and proactive innovation behavior. Specifically, mentors’ innovation support indirectly influences the proactive innovation behavior of postgraduates through the chain mediating effect of innovation efficacy and creative process engagement. Future research could explore these pathways across various innovative teaching practices, mentor guidance methods, and cultural contexts to further elucidate how mentors’ innovation support fosters the proactive innovation behavior of postgraduates at different developmental stages.

## Implications

First, it is essential to organize specialized training for mentors and cultivate a cultural atmosphere conducive to scientific research and innovation. Establishing a comprehensive mentor innovation support system is crucial for stimulating the active innovative behaviors of postgraduate students. By implementing targeted training for mentors and promoting an organizational culture of innovation, the initiative and quality of mentor support can be significantly improved. To this end, a specialized training mechanism should be developed to enhance mentors’ capabilities in providing innovation support. This can be achieved through special lectures and case workshops that focus on the primary objective of “shaping the role of supportive mentors.” Concurrently, efforts must be directed toward fostering an inclusive academic environment that encourages knowledge sharing between faculty and students, thereby enhancing postgraduate students’ awareness of their mentors’ innovative support. By facilitating deeper communication between mentors and students and addressing the relational needs of postgraduate students, this approach will empower them to engage in proactive innovation. Conversely, it is essential to foster an innovative culture at the organizational level. As the leader of the academic team, the mentor should proactively modify the evaluation criteria, shifting the emphasis from the outcomes of innovative achievements to the exploratory value inherent in the innovation process. Furthermore, mentors must fully encourage and trust the innovative endeavors of postgraduate students. When these students face challenges in their innovation efforts, mentors should intervene promptly to provide support, help clarify the core issues and underlying principles, and guide them in transforming their experiences of failure into valuable resources for future innovation activities.

Secondly, the intrinsic motivation of postgraduates should be stimulated, and their sense of innovation efficacy should be enhanced. The internal drive for innovation must be enhanced. Mentors should not only focus on the outcomes of innovations, but also on the growth experience during the innovation process. Given this, a sound process evaluation system must be established, thus setting different types of challenging goals for postgraduates, building a platform for recognition and sharing of innovative achievements, stimulating postgraduates’ intrinsic motivation, and continuously enhancing their sense of innovation efficacy. Positive self-awareness must be shaped. Mentors should share their innovative achievements with postgraduates. Additionally, mentors must guide students to recognize the value of innovation, explore their own advantages, and promote students’ identification of their own innovation role. At the same time, a supportive environment needs to be established, ensuring strong innovation resources, low innovation risk assumption, and high expectations for innovation results, to continuously improve the innovation efficacy of postgraduates.

Thirdly, attention must be paid to the innovation status of postgraduates, and creative process engagement must be strengthened. A monitoring mechanism of the innovation process must be established. Through the three-level monitoring mechanism of school-college-mentor, the innovation dynamics of postgraduates in universities should be accurately grasped, enabling targeted assistance measures to address weak links and guide students in overcoming obstacles. The process of scientific research innovation must be given high priority. Mentors should actively care about the level of innovation investment of their postgraduates, freeing them from tedious amateur activities and dedicating themselves wholeheartedly to innovation practice. At the same time, mentors should guide postgraduates to proactively connect themselves with information resources, help them explore solutions, and actively give feedback on students’ insufficient innovation, thus encouraging postgraduates to actively express their innovative ideas.

## Limitations and directions

This study investigates the relationship between mentors’ innovation support and the postgraduates’ proactive innovative behaviors; however, several limitations warrant consideration. First, while the research sample includes postgraduates of varying genders, disciplines, and academic levels, which facilitates the acquisition of diverse perspectives, the use of a random sampling method restricts the study’s scope to Jiangsu Province, China. This limitation may introduce selection bias, thereby compromising the representativeness of the sample and, consequently, the generalizability of the research findings.

Second, the research perspective needs to be deepened. Based on the conservation of resource theory and the social exchange theory, this study explores the mechanism of interaction between the mentors’ innovation support, postgraduates’ proactive innovative behaviors, innovative efficacy and creative process engagement. However, the emotional and behavioral responses that postgraduate students exhibit during the innovation process are often closely related to the organizational context they are in. Therefore, the influence of organizational-level factors on the postgraduates’ proactive innovative behaviors cannot be ignored either. Future research can further expand the theoretical framework, incorporate key antecedent variables such as organizational innovation atmosphere and psychological capital, and deeply explore their potential impact on the postgraduates’ proactive innovative behaviors, so as to enhance the integrity of the theoretical model.

Third, this study focuses solely on the intermediary roles of innovation efficacy and innovation process input in the relationship between mentor support and active innovation behavior. The research conclusions would benefit from further investigation into boundary conditions. Specifically, the organizational innovation atmosphere and individual differences may exert varying influences on this mechanism. Consequently, future research should explore the intermediary or moderating roles of additional factors in the context of mentors’ innovation support and the promotion of active innovation behavior.

Furthermore, data collection primarily depends on self-reporting methods. Although respondents are explicitly instructed to select the most accurate option in the questionnaire, the self-evaluation process may still be biased by the influence of perceived innovation efficacy and other subjective factors. Future research should incorporate multi-perspective data, such as evaluations from mentors and classmates, to assess postgraduates’ proactive innovation behavior, thereby enhancing the accuracy and objectivity of the measurement.

## Data Availability

The original contributions presented in the study are included in the article/Supplementary material, further inquiries can be directed to the corresponding author.
