# Supplementary material for: Influence of mentors’ innovation support on postgraduates’ proactive innovation behavior: the chain-mediating role of innovation efficacy and creative process engagement
Source: Front Psychol. 2026 Apr 29;17:1737206. doi: 10.3389/fpsyg.2026.1737206 (PMC13168191; doi:10.3389/fpsyg.2026.1737206)
Supplement: Supplementary file 1 [file Supplementary_file_1.pdf]

APPENDIX

Items for Primary Measures

Mentors' Innovation Support:

Please select the degree of compliance with the following statements based on your actual situation. ("completely inconsistent", "relatively inconsistent", "general", "relatively consistent", "completely consistent")

1. To foster my creative endeavors, my mentor diligently provides the necessary resources.
2. My mentor exemplifies a strong role model for creativity.
3. My mentor has publicly acknowledged my innovative efforts.
4. My mentor has motivated me to establish goals for innovation.
5. My mentor has bolstered my confidence in my creative potential.

Proactive Innovation Behavior

The following items describe the actions taken when completing a task or solving a problem in your research. Please select the degree of conformity based on the actual situation. ("completely inconsistent", "relatively inconsistent", "general", "relatively consistent", "completely consistent")

1. I keenly identified the problems that needed improvement in my work.
2. To solve the problem, I took the initiative to put forward suggestions.
3. I have a strong interest in innovation and truly desire it from the bottom of my heart.
4. I proactively seek out new ideas to improve work processes or products.
5. I welcome the collision of different ideas.
6. I think others' suggestions are valuable.
7. Before innovating, I try to find relevant resources (technology, funds, personnel, information) for support.
8. Before innovating, I look for similar examples of success or failure and explore the reasons behind them.
9. Before innovating, I thoroughly analyze the feasibility of various plans.

10. I dare to take risks in innovation.
11. I'm not afraid of failure in innovation.
12. I think success might follow failure.
13. I strive to overcome the difficulties encountered in the process of innovation.
14. When I encounter difficulties, I try to find ways to solve them instead of running away.
15. I have been trying out various options.
16. During the process of innovation, I firmly believe that the goal will definitely be achieved.

Innovation Efficacy

Please select the degree of compliance with the following statements based on your actual situation. (“completely inconsistent”, “relatively inconsistent”, “general”, “relatively consistent”, “completely consistent”)

1. I will be able to achieve most of the goals that I have set for myself in a creative way
2. When facing difficult tasks, I am certain that I will accomplish them creatively
3. In general, I think that I can obtain outcomes that are important to me in a creative way
4. I believe I can succeed at most any creative endeavor to which I set my mind
5. I will be able to overcome many challenges creatively
6. I am confident that I can perform creatively on many different tasks
7. Compared to other people, I can do most tasks very creatively
8. Even when things are tough, I can perform quite creatively

Creative Process Engagement

Respondents answered the following question: “In your job, to what extent do you engage in the follow actions when seeking to accomplish an assignment or solve a problem?” (“completely inconsistent”, “relatively inconsistent”, “general”, “relatively consistent”, “completely consistent”)

Problem identification:

1. I spend considerable time trying to understand the nature of the problem.
2. I think about the problem from multiple perspectives.
3. I decompose a difficult problem/assignment into parts to obtain greater understanding.

Information searching and encoding:

4. I consult a wide variety of information.

5. I search for information from multiple sources (e.g., personal memories, others' experience, documentation, Internet, etc.).

6. I retain large amounts of detailed information in my area of expertise for future use.

Idea generation:

7. I consider diverse sources of information in generating new ideas.

8. I look for connections with solutions used in seeming
diverse areas.

9. I generate a significant number of alternatives to the same problem before I choose the final solution.

10. I try to devise potential solutions that move away from established ways of doing things.

11. I spend considerable time shifting through information that helps to generate new ideas.
